# Childhood adversity and increased cytokine release from peripheral mononuclear cells of CHD patients – a pilot study

**DOI:** 10.1186/s12872-026-05713-z

**Published:** 2026-03-19

**Authors:** Henning Wiche, Ole Anhuef, Stefanie Martinache, Joe J. Simon, Norbert Frey, Johannes Backs, Hans-Christoph Friederich, Jobst-Hendrik Schultz, Bastian Bruns

**Affiliations:** 1 Department of General Internal Medicine, Psychosomatics, and Psychotherapy, Heidelberg University Hospital, Heidelberg, Germany; 2 Department of Cardiology, Angiology and Pneumology, Heidelberg University Hospital, Heidelberg, Germany; 3 Institute of Experimental Cardiology, Heidelberg University Hospital, Heidelberg, Germany; 4 DZHK (German Centre for Cardiovascular Research), Partner Site, Heidelberg/Mannheim, Heidelberg, Germany

**Keywords:** childhood adversity, early life stress, cardiovascular disease, cytokine release, PBMC, Inflammation

## Abstract

**Background:**

Early life stress (ELS) is a risk factor for cardiovascular disease. Chronic low-grade inflammation has been linked to cardiovascular vulnerability. The interplay of ELS and inflammation in the context of coronary heart disease (CHD) remains elusive and was investigated in this prospective case control study.

**Methods:**

In this prospective case-control study, *n* = 34 elective CHD inpatients at Heidelberg University Hospital were included between 2021 and 2023 and assessed regarding ELS by the Early Trauma Inventory Self Report Short Form (ETI-SR-SF). Salivary and venous blood samples were taken from the patients before and after a 6-minutes walking test. Median split of the population based on the ETI-SR-SF total score divided patients into a control group (< 5 points, *n* = 19 patients, 42.11% female, age 68 ± 11.28 years) and an ELS group (≥ 5 points, *n* = 15 patients, 26.67% female, age 67 ± 9.74 years), which were analyzed accordingly.

**Results:**

There were no differences in routine inflammatory parameters, including CRP (4.4 ± 4.6 vs. 8.8 ± 8.6 mg/l), leukocytes (8.6 ± 3.2 vs. 7.4 ± 1.9 /nl) and procalcitonin (0.07 ± 0.04 vs. 0.07 ± 0.03 ng/ml) (Student’s T-test). Conversely, peripheral blood mononuclear cells (PBMCs) of ELS patients, showed significantly higher IL-1β mRNA expression upon walking compared to baseline (0.77 vs. 1.63-fold, *p* = 0.0459, Student’s T-test). Also, IL-6 release from ELS patient PBMCs was significantly increased at 24 h (6.8 ± 7.1 vs. 53.5 ± 62.6 pg/ml, *p* = 0.0168, Student’s T-test). Ex-vivo exposure of PBMCs to high catecholamines blunted IL-6 release with upregulation of nuclear receptor 4A3 (NR4A3) expression in control vs. ELS patients (2.53- vs. 1.74-fold, *p* = 0.034, ANOVA).

**Conclusions:**

Our results suggest a possible role of PBMCs in mediating inflammation in CHD patients with early childhood stress.

## Background

Early life stress (ELS) is defined as adverse experiences during childhood such as physical, sexual, and emotional abuse or neglect and associated with a high risk of psychiatric and cardiovascular diseases and increased mortality [1, 2]. In Germany up to 18% of the population state that they were affected by moderate to severe neglect and up to 4.3% by moderate to severe abuse in their childhood and youth [3]. In a large epidemiological study from 1998 in the US with over 9000 patients to retrospectively record the various types of childhood adversity before the age of 18, over 50% of respondents reported at least one childhood adverse experience [4]. There also seems to be a dose-response relationship between the extent of adversity and the later risk of leading causes of death in adulthood, especially coronary heart disease (CHD) [2, 4–6], which is the most common cause of death in European countries [7]. ELS has been associated with cardiometabolic outcomes through factors which can be categorized into three main domains: health behaviors, physiology, and psychological well-being [8, 9]. A pathophysiological framework attempting to bring together findings of ELS association with different organ systems is the neuroimmune network hypothesis by Nusslock and Miller [10]. It proposes that ELS leads to heightened cortico-amygdala sensitivity towards threatening stimuli. This coincides with low-grade inflammation and aggravated cardiovascular responses compared to controls. The sympathetic nervous system (SNS) via norepinephrine and hypothalamic-pituitary-adrenal (HPA) axis appear to be mediating this pathway. Also, the prefrontal cortex seems to regulate activity in the amygdala and childhood adversity appears to influence development of this circuitry leading to more aggressive responses to provocation or easily perceive situations as threatening. Over time chronically elevated levels of cortisol lead to glucocorticoid insensitivity of peripheral immunomodulating cells such as monocytes and thereby sustaining a low-grade inflammatory state. Furthermore, reward sensitivity is seemingly affected in a way that one can hypothesize childhood adverse experiences facilitate the development of behavioral health risk factors such as smoking or physical inactivity. In the end crosstalk of peripheral inflammation and neural networks plays a significant and further to be explored etiological role in a wide range of diseases ranging from psychiatric to cardiovascular. The framework of the allostatic load overload hypothesis by Danese & McEwen uses a similar approach in explaining how adverse childhood experiences influence health outcomes [11]. Commencing from the observation of smaller volume prefrontal cortices, elevated HPA axis activation and inflammation levels in subjects with ELS in children and adults alike, they assume that ELS initiates alterations in biological systems that are important for maintaining homeostatic balance but can result in cumulative strain (allostatic load/overload) over time.

In ELS patients, alterations in biological systems are manifold. An increased cortisol awakening response with reduced overall baseline cortisol could be shown [12]. In this regard, oxytocin acts as a feedback inhibitor of HPA axis drive, resulting in improved symptom recovery [13]. Underlining the relevance of inflammation in stress related disorders, enhanced spontaneous pro-inflammatory cytokine production such as interleukin-1β (IL-1β) and interleukin-6 (IL-6) by PBMCs was shown in patients suffering from posttraumatic stress disorder (PTSD) compared to healthy controls [14]. Simultaneously, IL-1β and IL-6 play a pivotal role as acute phase inflammatory proteins and in the pathogenesis of atherosclerosis [15, 16]. Regarding cardiovascular diseases, a correlation between severity of CHD per se and increased cytokine release from PBMCs was also observed [17] as well as systemic inflammation and cortisol resistance in depressed CHD patients [18]. Members of the NR4A orphan nucleus receptor family also seem to play a key role mediating inflammation. Specifically, NR4A3 has been shown to decrease inflammation after myocardial infarction via suppression of NF-κB signaling [19].

On the premise that psychological distress is associated with an increased risk for CHD [20], here we focus on patients with ELS and CHD as a particular vulnerable patient group and seek to clarify underlying pathophysiology and clinical characteristics by investigating the role of biomarkers, including PBMC-driven cytokine release. Drawing from forementioned frameworks by Nusslock, Miller, Danese and McEwen we hypothesize that inflammation and cytokine release in CHD patients with ELS differ compared to no-ELS. Our insights could potentially help to improve diagnostic and therapeutic options for CHD patients with ELS.

## Methods

### Study design and participants

This was a monocentric, unblinded, prospective, non-interventional, case-control study. The study was conducted in accordance with the Declaration of Helsinki and approved by the local ethics committee (Ethics Committee at the Medical Faculty of Heidelberg University, S-592/2021). Elective inpatients at Heidelberg university hospital with the diagnosis of CHD with self-reported high burden of childhood adversity were compared with a group with low self-reported ELS burden. Group allocation was based on the Early trauma inventory – Self Report – Short Form (ETI) questionnaire (see below). The primary endpoint was a change in cytokine release (IL-1β, IL-6) of PBMCs by patients with an ETI sum score ≥ 5 vs. an ETI sum score < 5. For subsequent analyses, participants were split into two groups: “High ETI” (ETI-SR total ≥ 5) and “Low ETI” (ETI-SR total < 5). This cut off ETI score was selected based on the median score of this sample versus the mean ETI score in past papers because of the presence of extreme ETI scores in the data. While some studies have reported a higher mean ETI, our cut-off falls in line with previously reported healthy patient samples tested using the short-form version of the tool (mean = 3.5, sd = 3.3^39^; mean = 2.68, sd = 2.55^40^) [21]. In addition, various secondary endpoints were analyzed, including salivary α-amylase, oxytocin, and cortisol as well as clinical routine laboratory measurements, clinical characteristics, and comorbidities. Study participants were examined in the period October 2021 - February 2023.

### Study sample

In patients with PTSD - a common consequence of ELS and therefore probably the most comparable diagnosis - a spontaneous hyperresponsiveness of PBMCs can be found regarding the release of IL-1β or IL-6 (e.g., IL-6 or IL-1β (F = 11.31, *n* = 35)) [14]. Based on these results we calculated a required size of *n* = 15 per group (sample number planning with Primer of Biostatistics 7.0, McGraw-Hill) with an anticipated drop-out rate of 10%, resulting in *n* = 34 study participants for an effect size of 1.1, α = 0.05 and a power of 80%. With this sample size, a difference in cell stimulation should be detectable and associations with clinical phenotypes could possibly be investigated. Elective patients were actively recruited during their stay on our psycho-cardiological ward. Upon admission a screening was carried out using multiple questionnaires (ETI, PHQ-D, SF-36). The Early trauma inventory – Self Report – Short Form (ETI) is a questionnaire by Bremner et al. consisting of 27 items, which assess physical, emotional, and sexual abuse, as well as general traumas [22].

### Study inclusion and procedure

Patients with elective admission to the psycho-cardiology ward Siebeck at the Centre of Internal Medicine at Heidelberg University Hospital were approached for study participation upon completing the psycho-cardiology wards standard set of psycho-social evaluation questionnaires (ETI, SF-36, PHQ-D) by a member of the study team. The indications for elective admission to the ward were mostly planned performance of coronary angiography for diagnostic purposes or follow-up. The inclusion criteria were defined by a diagnosis of CHD according to ICD-10, an age of at least 18 years, sufficient knowledge of the German language, and a written declaration of consent. Patients with acute circumstances or psychiatric illness, PTSD, acute infection, myocardial infarction in the last year, use of immunomodulatory drugs, renal insufficiency, or lack of informed consent were excluded. After informed consent patients were included in the study and the study procedure was conducted in the morning on a day without other procedures, e.g. stress-echocardiography or cardiac MRI.

The procedure started with a 30 min fasting phase. Drinking, eating, smoking, and chewing gum were not allowed to prevent contamination of sampling. This was followed by a resting phase A (30 min bed rest). After this 2 ml saliva and 47 ml blood were collected to extract PBMCs and quantify various laboratory parameters via the central laboratory of Heidelberg University Hospital (see below). This was followed by a 6-minute walking test with subsequent collection of 27 ml blood (3 × 9 ml EDTA tubes). Concluding the rest phase B, where the patients had to rest in bed for 60 min, 27 ml blood (3 × 9 ml EDTA tubes) were drawn after completion.

**Fig. 1:**
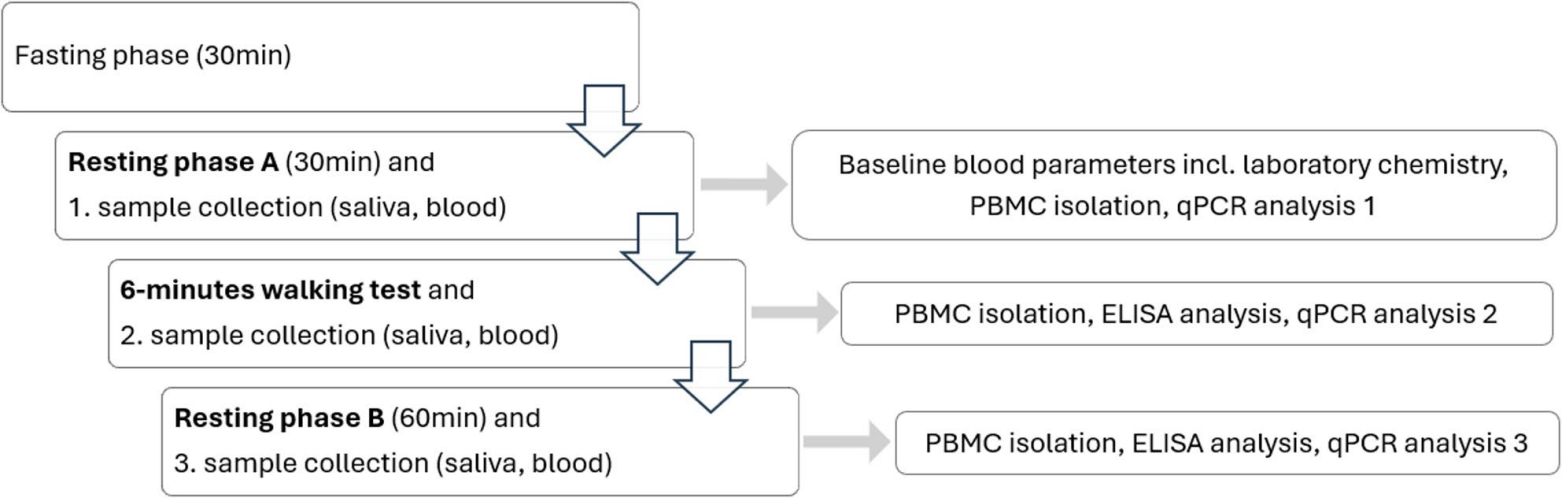
Study procedure

### Study measurements

#### Psychometric characterization: ETI, SF-36 and PHQ-D

Participation in the study was determined using the German version of the *Early Trauma Inventory Self Report – Short Form* (ETI). The ETI is a self-report questionnaire that retrospectively assesses adverse childhood experiences in the period from birth to age 18 in four categories. The first category consists of general adversity (11 items) such as natural disasters, serious accidents, illnesses, injuries, witnessing the death of another person, violence, and alcohol and drug abuse. The second consists of educational or physical punishment (5 items) such as being hit or kicked, burn injuries, injuries caused by objects being thrown, or the passive experience of being pushed or shoved. The third category consists of emotional adversity (5 items) including both emotional abuse and emotional neglect such as mockery, ignoring, or active devaluation or passive experiences of cold treatment or the feeling that one’s own needs are not understood by caregivers. The fourth category consists of sexual adversity (6 items) such as touching intimate parts of the body, rubbing genitals, coercing others to touch intimate parts of the body, and experiencing sexual intercourse, anal intercourse, or kissing without consent. In addition, two separate items ask about fear/horror/helplessness and/or dissociation in relation to the most subjectively serious experience reported in the ETI. The ETI is coded binarily. The items can be answered with “Yes” (1) or “No” (0). If the items marked with “Yes” (1) are added together, a maximum value of 27 points can be calculated for the overall scale. The ETI-SR-SF has demonstrated good psychometric properties, including satisfactory internal consistency and convergent validity in clinical samples [22–25].

In addition to the ETI, participants completed supplementary questionnaires collecting sociodemographic information such as gender, ethnicity, educational background, and current employment status to better describe the study sample. The written survey also included the German adaptations of the *Patient Health Questionnaire* (PHQ-D) and the *Short Form-36 Health Survey* (SF-36). The PHQ-D assesses psychological symptoms, including depressive disorders, anxiety disorders, somatoform complaints without identifiable organic causes, individual stress factors, alcohol misuse, and psychosocial functioning [26]. The SF-36 is a health-related instrument for evaluating quality of life, addressing domains such as physical functioning, role limitations due to physical or emotional problems, general health perceptions, vitality, social functioning, and mental well-being. For analysis, these domains can be grouped into the broader categories of mental health, physical health, and vitality [27].

#### 6 min walking test and BORG scale

The 6 min walking test is used in clinical practice to quantify chronic heart and lung diseases and was conducted according to the SOP of the German Center for Cardiovascular Research (DZHK-SOP-K-04 V1.0) [28]. In this study, it was primarily used as a stressor to stimulate physical stress reactions. The patients were instructed to not undertake any physical activity two hours before the test. Walking aids, which are also used in everyday life, were permitted during the test and oxygen administration was continued, if needed before. The absolute contraindications were extended to include the relative contraindications as well: stable and instable angina pectoris, myocardial infarction in the past month, a resting heart rate > 120/min, a systolic blood pressure > 180 mmHg, a diastolic blood pressure > 100 mmHg. They were reviewed prior to study inclusion. After a 5 min rest, blood pressure, pulse and oxygen saturation were measured. Afterwards, the patients completed the standardized BORG scale (see below) to measure subjectively perceived shortness of breath. The patients were then instructed about the test procedure. This involved the patient walking back and forth as quickly as possible between two markers for 6 min and this was demonstrated to them. In case of exhaustion, breaks were allowed, and the test could be continued after recovery. The test was discontinued, if one of the following symptoms occurred: angina pectoris, severe shortness of breath, dizziness, unsteady gait, risk of falling, calf cramps, claudicatio, SpO2 < 90%. After completion of the walking test, shortness of breath and exertion were again assessed using the BORG scale. Blood pressure, pulse and oxygen saturation were measured.

#### Salivary cortisol, -oxytocin and -α-amylase measurements

Saliva was collected using 2 × 1 ml SaliCaps^®^ (Tecan IBL International) at three points in time (see Fig. 1) for analysis of cortisol, oxytocin and α-amylase. The measurement was carried out by the Institute of Medical Psychology at Heidelberg University Hospital after all study participants had been included in a blinded fashion. In the period between sample collection and measurement, saliva samples were stored at -80 °C. The measurements were carried out using an ELISA for cortisol (Cortisol free in saliva ELISA by Demeditec Diagnostics GmbH) and oxytocin (Oxytocin ELISA Kit by Enzo Life Sciences) and an enzymatic photometric test for α-amylase (α-amylase CC FS by DiaSys Diagnostic Systems).

#### Routine laboratory measurements

The blood samples taken with a Sarstedt Safety-Multifly^®^ cannula, 21G x 3/4’’ at a peripheral vein before the walking test were sent to the central laboratory of the Heidelberg University Hospital via in-house channels for analysis. These contained a total of 20.4 ml whole-blood in a serum gel, heparin, EDTA and citrate coagulation tube. The laboratory parameters being quantified were procalcitonin (PCT), aldosterone, C-reactive protein (CRP), differential blood count, osteocalcin, ferritin, TSH/fT3/fT4, copeptin, NTproBNP, hs-TnT, as well as Quick/INR.

#### PBMC isolation and experiments

The blood samples were processed at the Institute for Experimental Cardiology at Heidelberg University Hospital, beginning with the isolation of peripheral blood mononuclear cells (PBMCs) immediately after retrieval using PBMC spin medium (pluriSelect) to form a density gradient by centrifugation. For this purpose, the blood of three EDTA tubes (9 ml each) collected at each sample collection were diluted 1:1 with phosphate-buffered saline (PBS). The PBS-blood mixture was then carefully added to 15 ml of PBMC spin medium without mixing the phases, as this has a cytotoxic effect. After centrifugation, a density gradient formed based on the specific density of the PBMC spin medium. Plasma and thrombocytes were located at the top, lymphocytes and monocytes (PBMCs) in the interphase, the spin medium below, and erythrocytes with a few granulocytes in the bottom layer. In cell culture plates, the isolated PBMCs were treated with epinephrine hydrochloride (EPI, Sanofi Aventis, 500 µM) for a period of 24 h for the determination of cytokine release from supernatant and for a period of 1 h for mRNA analysis. The measurement was conducted with 2 million PBMC per batch stimulated with epinephrine (500 µM) in Dulbecco’s Modified Eagle’s Medium – high glucose (DMEM) (Sigma) or medium without epinephrine as control. This was done in triplicates or quadruplicates depending on the number of PBMCs available. The dose of 500 µM was chosen to mimic excessive catecholaminergic stress. High doses of EPI have been used successfully in vivo to mimic stress-induced acute heart failure as in Takotsubo syndrome [29]. After the stimulation period of one hour, the supernatant fluid was removed from all wells and centrifuged to remove the cells. This was stored at -80 °C until further analysis. Furthermore, PBMCs were removed from the wells, transferred to Eppendorfer reaction tubes and centrifuged. Purification was conducted. The cell pellet was stored at -80 °C until RNA isolation. After the stimulation period of 24 h, supernatant fluid was removed from all wells, purified, and stored at -80 °C.

#### Flow cytometry analysis

3 × 1 million PBMCs were used for analysis. Flow cytometry was performed using the FACSVerse flow cytometer (BD Biosciences). The PBMCs were stained in several steps. 3 × 1 million unstimulated PBMC controls were washed and afterwards 5 µl of Fc receptor blocker was added to prevent non-specific antibody binding and incubated for 10 min at room temperature. The third sample served as an unstained control. Without further washing, either fluorochrome-conjugated antibodies for sevenfold staining or isotype controls (IgG1 FITC and APC) were added and incubated for 30 min at 4 °C. This was followed by purification through washing three times with FACS buffer and centrifugation. The cells were resuspended in 500 µl FACS buffer (1 million cells/500 µl) and analyzed directly afterwards. The following antibodies (Biolegend) were used for staining.

**Table 1:** Monoclonal antibodies used for flow cytometry with corresponding fluorochrome conjugation

Antibody	Fluorochrome
CD196	Brilliant Violet 721
CD14	FITC
CD8	PE
CD16	APC
CD19	PerCP-Cy5.5
CD3	APC-Cy7
CD183	PE-Cy7

*CD C*luster of differentiation, *FITC* Fluorescein isothiocyanate, *PE* Phycoerythrin, *APC* Allophycocyanin, *PerCP-Cy5.5 *Peridinin-chlorophyll-protein conjugated to cyanine 5.5, *APC-Cy7* Allophycocyanin conjugated to cyanine 7, *PE-Cy7* Phycoerythrin conjugated to cyanine 7

Compensation was performed automatically by the FACSuite software, with BD Research Beads being read in before each measurement to calibrate the lasers. The evaluation and identification of the various cell populations of the PBMCs was performed using FlowJo (version 10.8). After excluding cell debris, the following gating strategy was used to identify the subpopulations. When CD3 + cells were applied against CD19 + cells, the B lymphocytes (CD19+) could be separated, whereas the CD3 + population represented the T lymphocytes. The CD3-/CD19- population represented the monocytes. These were further specified into the CD14+, CD16+, and CD14+/CD16 + monocyte groups using CD14 and CD16. The T lymphocytes (CD3+) were further separated into cytotoxic lymphocytes (CD8+), as well as the CD8- population representing CD4 + lymphocytes, also known as T helper (Th) lymphocytes. The CD4 + lymphocytes could be further subdivided by applying CD183 + vs. CD196+. The CD183 + population represented the Th1 lymphocytes, while the CD193 + population defined the Th17 lymphocytes. The CD4 + lymphocytes that were both CD183- and CD193- represented the Th2 lymphocytes.

#### RNA isolation and qPCR

Total RNA was isolated from PBMCs using TRIzol (Invitrogen). After treating the RNA with DNase, 1 µg was used for cDNA synthesis with the first strand reverse transcription kit (Steinbrenner) and the PCR-Thermocycler (Modell Mastercycler X50i by Eppendorf) for qRT-PCR. Quantitative PCR (qPCR) was performed using the PowerUp™ SYBR™ Green Mastermix (ThermoFisher Scientific), with detection carried out on a real-time PCR cycler (LightCycler^®^ 480 II) (Roche). The following primer sequences were used for this purpose.

**Table 2:** Primer sequences

Gene		Sequence
NR4A1	Forward	GGACAACGCTTCATGCCAGCAT
	Reverse	CCTTGTTAGCCAGGCAGATGTAC
NR4A2	Forward	AAACTGCCCAGTGGACAAGCGT
	Reverse	GCTCTTCGGTTTCGAGGGCAAA
NR4A3	Forward	ACTGCCCAGTAGACAAGAGACG
	Reverse	GTTTGGAAGGCAGACGACCTCT
CCL2	Forward	CAGCAAGTGTCCCAAAGAAGC
	Reverse	TCGGAGTTTGGGTTTGCTTG
IL-6	Forward	AGACAGCCACTCACCTCTTCAG
	Reverse	TTCTGCCAGTGCCTCTTTGCTG
IL-1β	Forward	CAGAAGTACCTGAGCTCGCC
	Reverse	AGATTCGTAGCTGGATGCCG
GAPDH	Forward	ACCCACTCCTCCACCTTTGAC
	Reverse	ACCCTGTTGCTGTAGCCAAATT

*NR4A1* Nuclear receptor 4a1, *NR4A2* Nuclear receptor 4a2, *NR4A3* nuclear receptor 4a3, *CCL2* C-C-motif chemokine ligand 2, *IL-6* Interleukin 6, *IL-1β* Interleukin 1β, *GAPDH* Glycerinaldehyd-3-phosphat-dehydrogenase

#### Enzyme-linked immunosorbent assay (ELISA)

The measurement of interleukin (IL)-6 and interleukin (IL)-1β were carried out from the supernatant of patient PBMCs using the Human IL-1β and IL-6 ELISA kit (ThermoFisher Scientific). Duplicate measurements were carried out for each sample, according to the manufacturer´s protocol. Absorbance was measured at 450 nm with an ElisaReader (EnSpire^®^ by PerkinElmer).

### Statistics

Clinical data are presented as mean ± standard deviation (SD) for continuous variables with a normal distribution and median (interquartile range, IQR) for continuous variables with a non-normal distribution (Shapiro–Wilk test) or as amount (n) and frequency (%) for categorical variables. Missing data were not imputed. Student’s t-test and Mann–Whitney U test were used to compare continuous variables with normal and non-normal distributions, respectively. Chi-squared test was used to compare categorical variables. Experimental data (qPCR, ELISA) are expressed as mean ± SEM. Normal or lognormal distribution was verified by the Kolmogorov–Smirnov test. Statistical analysis included one-way ANOVA or Kruskal–Wallis test followed by Sidák’s post-hoc test. An unpaired or paired Student’s t-test or Mann–Whitney U test were used when appropriate. A *p* < 0.05 was considered statistically significant. Statistical analysis and creation of figures was carried out using SPSS Statistics 29.0.2.0 by IBM, Graph Pad Prism 10 by GraphPad Software, and Biorender [30].

## Results

### Descriptive statistics of the study population

**Fig. 2:**
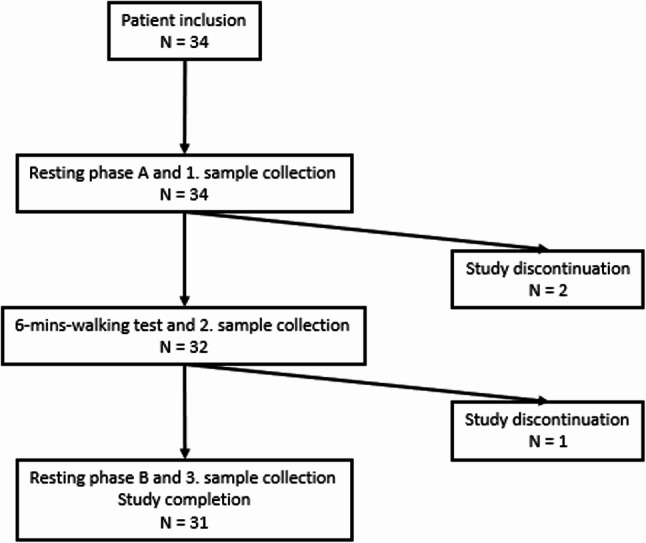
Flowchart of patient inclusion

A total of *n* = 34 patients were examined consisting of a control (< 5 points, *n* = 19 patients, 42.11% female, age 68 ± 11.28 years) and ELS group (≥ 5 points, *n* = 15 patients, 26.67% female, age 67 ± 9.74 years) (Fig. 2).

Compared to the control group, there was a significantly higher incidence of pulmonary hypertension in the ELS group (0% vs. 26.7%, *p* < 0.017). There was no significant difference in other comorbidities, such as 3-vessel CHD (36.9% vs. 53.3%), heart failure (26.3% vs. 13.3%), diabetes (15.7% vs. 33.3%) or COPD (10.5% vs. 20%). There was a significantly higher use of l-thyroxine (0% vs. 33.3%, *p* < 0.006) and insulin (0% vs. 20.0%, *p* < 0.041) in the ELS group (Chi^2^ test). Regarding other medication as wells as cardiovascular risk factors no significant differences were observed (Table 3).

**Table 3:** Clinical characteristics of the study population

Characteristics	ETI < 5 (*N* = 19)		ETI ≥ 5 (*N* = 15)		*P*	
	*n*, mean, median	%, SD, IQR	*n*, mean, median	%, SD, IQR		
Sociodemographic						
Age	68.32	11.28	67.33	9.74	0.791	
Male	11	57.9	11	73.3	0.350	
Female	8	42.1	4	26.7	0.350	
European	19	100.00	13	86.7	0.101	
Asian	0	0.00	2	13.3	0.101	
High School	2	10.5	2	13.3	0.801	
< High School	11	57.9	8	53.3	0.790	
University	3	15.8	3	20.0	0.749	
Other Education	3	15.8	2	13.3	0.841	
Employed	5	26.7	6	40.1	0.397	
Not Employed	2	10.5	1	6.7	0.694	
Retired	9	47.5	7	46.7	0.968	
Other Employment	3	15.8	1	6.7	0.412	
Cardiovascular diseases / risk factors						
Arterial hypertension	16	84.2	13	86.7	0.841	
Current nicotine abuse	2	10.5	2	13.3	0.801	
Ex nicotine abuse	13	68.4	7	46.7	0.201	
Cumulative pack years	23.11	21.04	28.08	30.31	0.587	
Hypercholesterolemia	12	63.2	12	80.0	0.285	
Diabetes mellitus II	3	15.8	5	33.3	0.231	
Diabetes mellitus I	0	0.0	1	6.7	0.253	
Obesity	5	26.3	6	40.0	0.397	
Pulmonary hypertension	0	0.0	4	26.7	0.017*	
Heart failure	5	26.3	2	13.3	0.353	
Atrial fibrillation	7	36.8	4	26.7	0.529	
Dilated CMP	1	5.3	1	6.7	0.863	
Hypertrophic CMP	1	5.3	0	0.0	0.367	
History of myocardial infarction	3	15.8	2	13.3	0.841	
PAD	3	15.8	4	26.7	0.436	
CVI	0	0.0	2	13.3	0.101	
COPD	2	10.5	3	20.0	0.439	
Cardiovascular parameters of the 6 min. walking test						
* Baseline*						
SpO2 [%]	95	3.03	95	2.83	0.916	
Systolic RR [mmHg]	133	22.16	130	15.76	0.626	
Diastolic RR [mmHg]	74	15.9	76	12.9	0.880	
Heart rate [/min]	64	10.22	72	9.46	0.026*	
BORG-scale of dyspnea	0.31	0.546	1.04	1.781	0.110	
* After walking*						
SpO2 [%]	95	5.21	96	2.32	0.421	
Systolic RR [mmHg]	144	18.47	139	30.33	0.547	
Diastolic RR [mmHg]	81	15.17	82	15.02	0.879	
Heart rate [/min]	76	13.72	79	13.92	0.561	
BORG-scale of dyspnea	2.47	1.913	1.75	2.200	0.329	
B ORG-scale of exertion	12.06	3.226	11.5	2.876	0.616	
Medication						
ASS	7	36.8	8	53.3	0.336	
Beta-blockers	11	57.9	10	66.7	0.601	
ACE-inhibitors / ARBs	16	84.2	11	73.3	0.436	
Calcium channel blockers	3	15.8	7	46.7	0.057	
Mineralcorticoid receptor antagonist	5	26.3	2	13.3	0.353	
Other diuretics	9	47.4	9	60.0	0.464	
Statins	11	57.9	12	80.0	0.171	
Anticoagulation	14	73.7	7	46.7	0.107	
Antidepressants	2	10.5	1	6.7	0.694	
Benzodiazepine	0	0.0	1	6.7	0.253	
Z substances	1	5.3	1	6.7	0.863	
Dopamine agonist	2	10.5	1	6.7	0.694	
Opioids	1	5.3	2	13.3	0.410	
Proton-pump inhibitors	5	26.3	8	53.3	0.107	
L-thyroxine	0	0.0	5	33.3	0.006*	
Antidiabetics	5	26.3	4	26.7	0.982	
Insulin	0	0.0	3	20.0	0.041*	
Gabapentinoids	2	10.5	1	6.7	0.694	
Inhaled corticosteroids as needed	4	21.1	3	20.0	0.940	
Inhaled anticholinergic as needed	4	21.1	4	26.7	0.702	

*ETI* Early Trauma Inventory Self Report – Short Form, *CMP* Cardiomyopathy, *PAD* peripheral artery disease, *CVI*Chronic venous insufficiency, *COPD* Chronic obstructive pulmonary disease, *RR* Blood pressure, *SpO2 *Oxygen saturation in the blood, *ASS* Acetylsalicylic acid, *ACE* Angiotensin-converting enzyme, *ARB* Angiotensin receptor blocker

*P < 0.05 (by Chi², unpaired Student´s t-test or Mann-Whitney-U-test)

Laboratory chemistry showed no significant differences in inflammatory parameters such as CRP (8.8 ± 8.6 vs. 4.4 ± 4.6 mg/l), leukocytes (7.4 ± 1.9 vs. 8.6 ± 3.2 /nl) and procalcitonin (0.7 ± 0.3 vs. 0.07 ± 0.4 ng/ml) (Student’s T-test). Furthermore, there was no significant difference in the number of cell populations from the differential in our data (Ps > 0.05, Table 4). The ELS group showed an increased resting heart rate before the walking test (72/min vs. 63/min, *p* = 0.026) (Table 3).

**Table 4:** Baseline blood parameters

Characteristics	ETI < 5 (*N* = 19)		ETI ≥ 5 (*N* = 15)		*P*	
	mean or median	SD or IQR	mean or median	SD or IQR		
Leucocytes [/nl]	8.58	3.24	7.42	1.88	0.229	
Erythrocytes [pl]	4.61	0.58	4.72	0.77	0.621	
Thrombocytes [/nl]	240.63	57.88	230.13	50.1	0.582	
Neutrophiles [/nl]	6.52	3.31	5.01	1.69	0.118	
[%]	73.58	11.89	66.57	8.09	0.06	
Lymphocytes [/nl]	2.14	2.25	1.54	0.59	0.322	
[%]	16.5	10.1	21.65	7.66	0.112	
Monocytes [/nl]	0.47	0.17	0.51	0.19	0.496	
[%]	6.04	2.36	6.87	2.16	0.298	
Eosinophiles [/nl]	0.15	0.12	0.22	0.21	0.212	
[%]	2.04	1.59	3.03	2.74	0.202	
Basophiles [/nl]	0.034	0.015	0.032	0.021	0.771	
[%]	0.43	0.22	0.44	0.30	0.943	
CRP [mg/l]	4.44	4.60	8.77	8.56	0.068	
TropT [pg/ml]	31.47	29.1	29.8	20.14	0.853	
Procalcitonin [ng/ml]	0.06974	0.04123	0.06900	0.02548	0.952	
NT-proBNP [ng/l]	1699.95	2198.46	898.13	1533.18	0.239	
Ferritin [µg/l]	135.47	164.31	135.33	110.23	0.998	
Glucose [mg/dl]	157.83	93.59	176.47	97.26	0.580	

*ETI* Early Trauma Inventory Self Report – Short Form, *CRP* C- reactive peptid, *TropT *Troponin T, *NT-proBNP* Terminal pro natriuretic peptide

*P < 0.05 by unpaired Student´s t-test or Mann-Whitney-U-test

Flow cytometry analysis revealed no significant differences regarding the PBMC subpopulations between both groups (Table 5).

**Table 5:** PBMC subpopulations per 1 million PBMCs divided by flow cytometry

PBMC subpopulation	ETI < 5 (*N* = 17–19)		ETI ≥ 5 (*N* = 14–15)		*P*	
	mean	SD	mean	SD		
First sample collection						
Lymphocytes	19610.84	7524.652	22211.73	6982.926	0.310	
B-lymphocytes	2952.05	2077.173	3123.80	1474.356	0.788	
T-lymphocytes	16658.79	7112.255	19087.93	6210.076	0.304	
CD4-T-lymphocytes	10847.74	4817.192	12752.40	4780.505	0.259	
CD4-TH1-lymphocytes	2596.74	1292.526	3096.20	1419.023	0.292	
CD4-TH2-lymphocytes	5068.11	2217.456	5377.27	2419.800	0.701	
CD4-TH17-lymphocytes	1450.68	1173.228	1941.53	1007.093	0.207	
CD8-T-lymphocytes	4437.84	3162.473	6312.93	3251.389	0.100	
Monocytes	9601.63	5566.022	9759.47	3178.135	0.923	
CD14-monocytes	827.47	952.770	955.80	870.432	0.688	
CD16-monocytes	3105.32	1953.066	4312.53	3572.543	0.218	
CD14/CD16-monocytes	472.11	373.444	812.80	653.715	0.065	
Second sample collection						
Lymphocytes	18329.56	7490.957	22720.14	4985.756	0.069	
B-lymphocytes	2945.44	2504.602	3295.57	1431.431	0.644	
T-lymphocytes	15384.11	7101.541	19424.57	4493.296	0.073	
CD4-T-lymphocytes	10714.44	4812.561	13279.00	3957.953	0.117	
CD4-TH1-lymphocytes	2728.17	1355.264	3569.43	1558.356	0.113	
CD4-TH2-lymphocytes	4903.33	2230.152	5838.07	1953.630	0.224	
CD4-TH17-lymphocytes	1433.61	1155.641	1632.71	686.859	0.573	
CD8-T-lymphocytes	4672.89	3375.010	6111.21	2893.836	0.213	
Monocytes	11688.44	6453.832	10930.79	3147.891	0.690	
CD14-monocytes	590.50	418.598	989.00	858.785	0.094	
CD16-monocytes	3898.56	2370.539	5246.07	3678.197	0.218	
CD14/CD16-monocytes	579.44	494.350	865.36	565.551	0.138	
Third sample collection						
Lymphocytes	19130.76	7676.130	23350.36	6876.545	0.121	
B-lymphocytes	3091.47	2184.270	3319.00	1280.997	0.734	
T-lymphocytes	16039.29	7067.982	20031.36	6263.415	0.111	
CD4-T-lymphocytes	11301.06	5334.361	13893.50	4918.054	0.174	
CD4-TH1-lymphocytes	2851.59	1603.751	3768.86	1587.241	0.122	
CD4-TH2-lymphocytes	4915.76	2535.548	5574.43	2206.366	0.452	
CD4-TH17-lymphocytes	1586.82	1070.814	1826.07	763.185	0.489	
CD8-T-lymphocytes	4715.38	2961.174	6104.14	3205.949	0.228	
Monocytes	9267.35	4523.472	9942.00	3415.657	0.649	
CD14-monocytes	642.71	483.334	1012.07	759.220	0.111	
CD16-monocytes	3241.65	1986.843	4223.00	3324.034	0.317	
CD14/CD16-monocytes	625.06	557.736	886.14	528.649	0.195	

*ETI* Early Trauma Inventory Self Report – Short Form, *CD* Cluster of differentiation, *TH* Helper cells, *B* bone marrow, *T* Thymus

*P < 0.05 by unpaired Student´s t-test

Regarding psychometric characterization there was a significant difference between the low-ELS vs. high-ELS group for the ETI total score after median split (9 ± 5.4 vs. 1.7 ± 1.5, *p* = 0.001), which was also reflected in the sub-scores. However, no significant differences could be observed regarding the characteristics of the PHQ-D and the SF-36 with respect to depression, anxiety, stress, as well as quality of life. However, there was a non-significant trend towards higher levels of reported stress and anxiety in ELS patients compared to the low-ELS group (Table 6).

**Table 6:** Psychometric characterization

Questionnaire with sub scales	ETI < 5 (*N* = 19)		ETI ≥ 5 (*N* = 15)		*P*	
	mean	SD	mean	SD		
ETI total	1.68	1.53	9.13	5.46	< 0.001**	
ETI general	1.05	1.27	3.07	2.6	0.006*	
ETI educational/physical	0.47	0.91	2.27	1.39	< 0.001**	
ETI emotional	0.11	0.46	1.93	1.75	< 0.001**	
ETI sexual	0.0	0.0	0.73	1.44	0.03*	
ETI trauma criteria	0.05	0.23	1.13	0.74	< 0.001**	
PHQ-D depression	4.19	3.75	6.26	4.67	0.186	
PHQ-D anxiety	2.47	1.84	5.14	5.17	0.056	
PHQ-D stress	3.46	2.6	5.7	3.52	0.055	
PHQ-D physical	8.43	5.92	11.14	5.79	0.233	
SF-36 mental health	49.8	11.83	47.41	12.97	0.635	
SF-36 physical health	38.78	11.38	35.22	11.09	0.436	
SF-36 vitality	56.08	21.41	44.64	24.69	0.178	

*ETI* Early Trauma Inventory – Self Report – Short Form, *PHQ-D* Patient Health Questionnaire, *SF-36* Short Form 36 Health Survey

*P < 0.05

**P < 0.001 by unpaired Student´s t-test

### Reduced salivary alpha amylase and cortisol in ELS patients

**Fig. 3:**
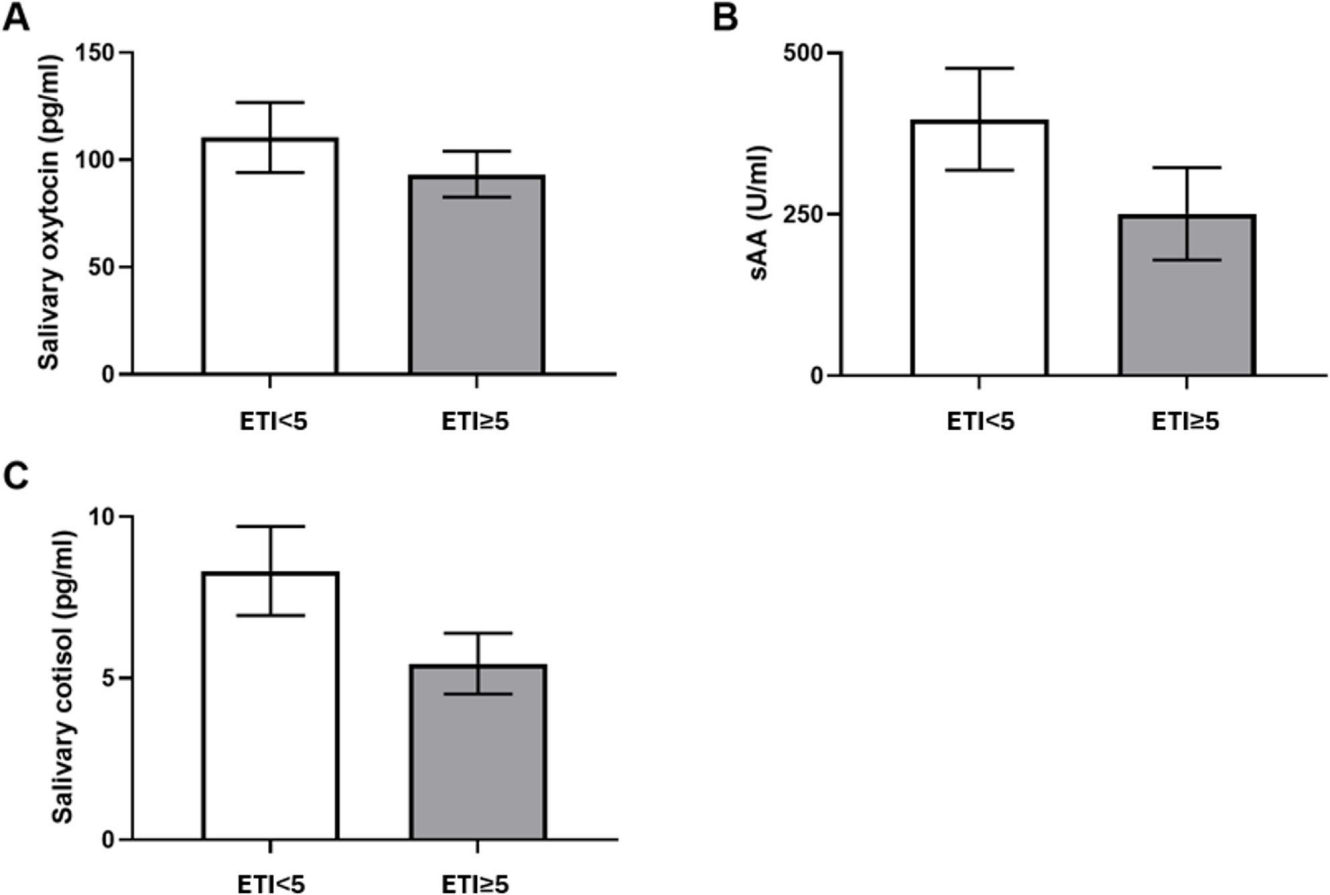
Baseline saliva analyses. Measured after a 30 min rest and fasting period using an enzyme-linked immunosorbent assay (ELISA). Comparison between control (ETI < 5) and ELS (ETI ≥ 5) group. **A** salivary oxytocin, N(ETI < 5) = 14, N(ETI ≥ 5) = 11, (**B**) salivary alpha-amylase (sAA), N(ETI < 5) = 17, N(ETI ≥ 5) = 12 , (**C**) salivary cortisol, N(ETI < 5) = 14, N(ETI ≥ 5) = 11, data shown as mean ± SD

Salivary samples for measuring of oxytocin could be collected successfully in 29 of 34 patients (85%). Mean oxytocin in the ETI < 5 group was 110.46 pg/ml, median 110.75 pg/ml and standard deviation 63.04. Mean oxytocin in the ETI ≥ 5 group was 120.91 pg/ml, median 96.42 pg/ml, and standard deviation 96.87. There was no significant difference between both groups (Fig. 3A). Samples for measuring of salivary alpha amylase (sAA) could be collected in 29 of 34 patients (85%). Mean sAA in the ETI < 5 group was 397.27 U/ml, median 412.14 U/ml and standard deviation 314.47. Mean in the ETI ≥ 5 group was 250.87 U/ml, median 125.37 U/ml and standard deviation 237.27. There was no significant difference between both groups (*p* = 0.2, t-test) (Fig. 3B). Salivary samples for measuring of cortisol could be collected in 26 of 34 patients (76%). Mean in the ETI < 5 group was 8.32 ng/ml, median 7.38 ng/ml, standard deviation 5.15. Mean in the ETI ≥ 5 group was 5.45 ng/ml, median 4.28 ng/ml, standard deviation 2.98. There was a trend towards lower saliva cortisol levels at baseline rest in ELS patients that was not significant (*p* = 0.07, Mann-Whitney test) (Fig. 3C). Taken together, there was a trend towards lower resting salivary cortisol and sAA in ELS patients.

### Elevated PBMC IL-1β expression and overall IL-6 release in ELS patients blunted by high-dose epinephrine

**Fig. 4:**
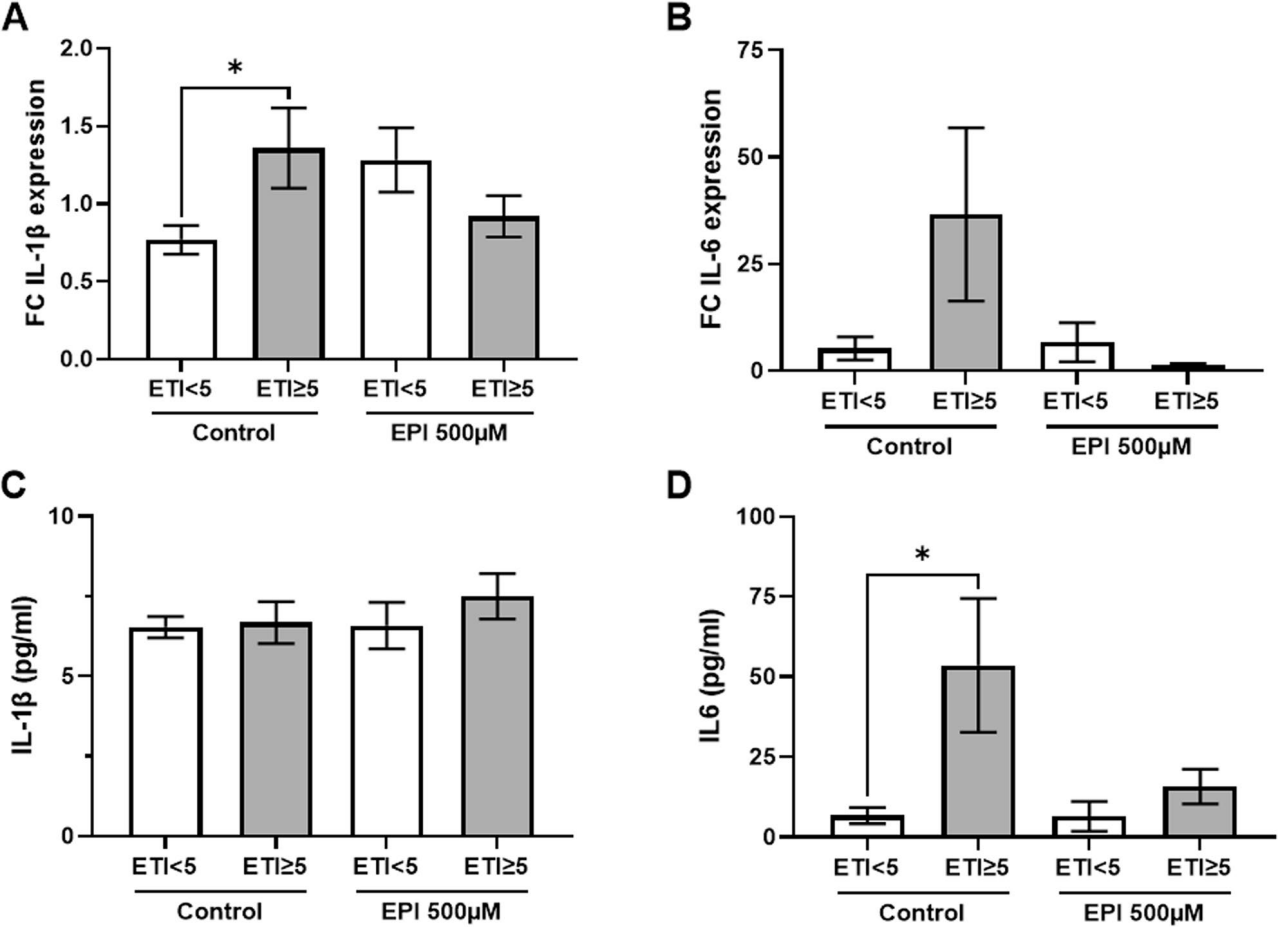
Elevated PBMC IL-1β expression and overall IL-6 release in ELS patients blunted by high-dose epinephrine. Fold-change (FC) interleukin-1β (IL-1 β) (**A**) and interleukin-6 (IL-6) (**B**) mRNA expression per gapdh between resting phase A and resting phase B in peripheral blood mononuclear cells (PBMCs) after 1 h of stimulation with either control medium (Control) or 500 µM epinephrine (EPI) from patients with coronary heart disease and a sum score < 5 vs. ≥ 5 in the Early Trauma Inventory self-report short-form (ETI). Overall IL-1 β (**C**) and IL-6 (**D**) in the supernatant of PBMCs cultured for 24 h either in Control or EPI at resting phase B grouped by ETI. N(A Control ETI < 5) = 17, N(A Control ETI ≥ 5) = 13, N(A EPI ETI < 5) = 16, N(A EPI ETI ≥ 5) = 12, N(B Control ETI < 5) = 16, N(B Control ETI ≥ 5) = 13, N(B EPI ETI < 5) = 16, N(B EPI ETI ≥ 5) = 12, N(C Control ETI < 5) = 9, N(C Control ETI ≥ 5) = 8, N(C EPI ETI < 5) = 8, N(C EPI ETI ≥ 5) = 9, N(D Control ETI < 5) = 8, N(D Control ETI ≥ 5) = 9, N(D EPI ETI < 5) = 8, N(D EPI ETI ≥ 5) = 9. ANOVA, *p < 0.05

PBMC fold-change expression of IL-1β increased significantly after the 6 min walking test in patients with an ETI ≥ 5 compared to patients with an ETI < 5. Incubation for 1 h with high-dose epinephrine – mimicking excessive stress – caused a slightly increased fold-change of IL-1β expression in ETI < 5 patients while not significantly affecting ETI ≥ 5 patients (Fig. 4A). In line with this finding the fold-change expression of IL-6 was increased in ETI ≥ 5 patients, which was blunted by high-dose EPI but not statistically significant (Fig. 4B). IL-1β (Fig. 4C) and IL-6 ELISA from PBMC supernatant after 24 h of incubation with or without EPI revealed a significantly higher IL-6 release from PBMCs of patients with an ETI ≥ 5 compared to an ETI < 5 (Fig. 4D). This effect was blunted by high-dose epinephrine. Also, linear regression showed that higher ETI SR-SF total scores were significantly associated with higher IL-6 levels (B = 3.63, 95% CI [0.08, 7.17], *p* = 0.046) and fold-change to recovery values for IL-1β (*B* = 0.067, 95% CI [0.000, 0.134], *p* = 0.050). In addition, in a multivariate regression analysis the observed difference for fold-change to recovery for IL-1β remained significant after adjustment for sex, hyperlipidemia, diabetes, obesity, and NT-proBNP (*B* = 0.91, *p* = 0.012), with the full model reaching statistical significance (*F*(6, 27) = 2.80, *p* = 0.030). Among the covariates, obesity was independently associated with lower fold-change values (*B* = − 0.92, 95% CI [− 1.70, − 0.14], *p* = 0.023). For IL-6 the group difference did not persist after adjustment for above mentioned covariates. Interestingly, EPI did not yield any impact on 24 h total IL-1β release from PBMCs of patients scoring ETI ≥ 5 or ETI < 5.

### Blunted high-dose epinephrine-induced upregulation of PBMC nuclear receptor expression in ELS patients

**Fig. 5:**
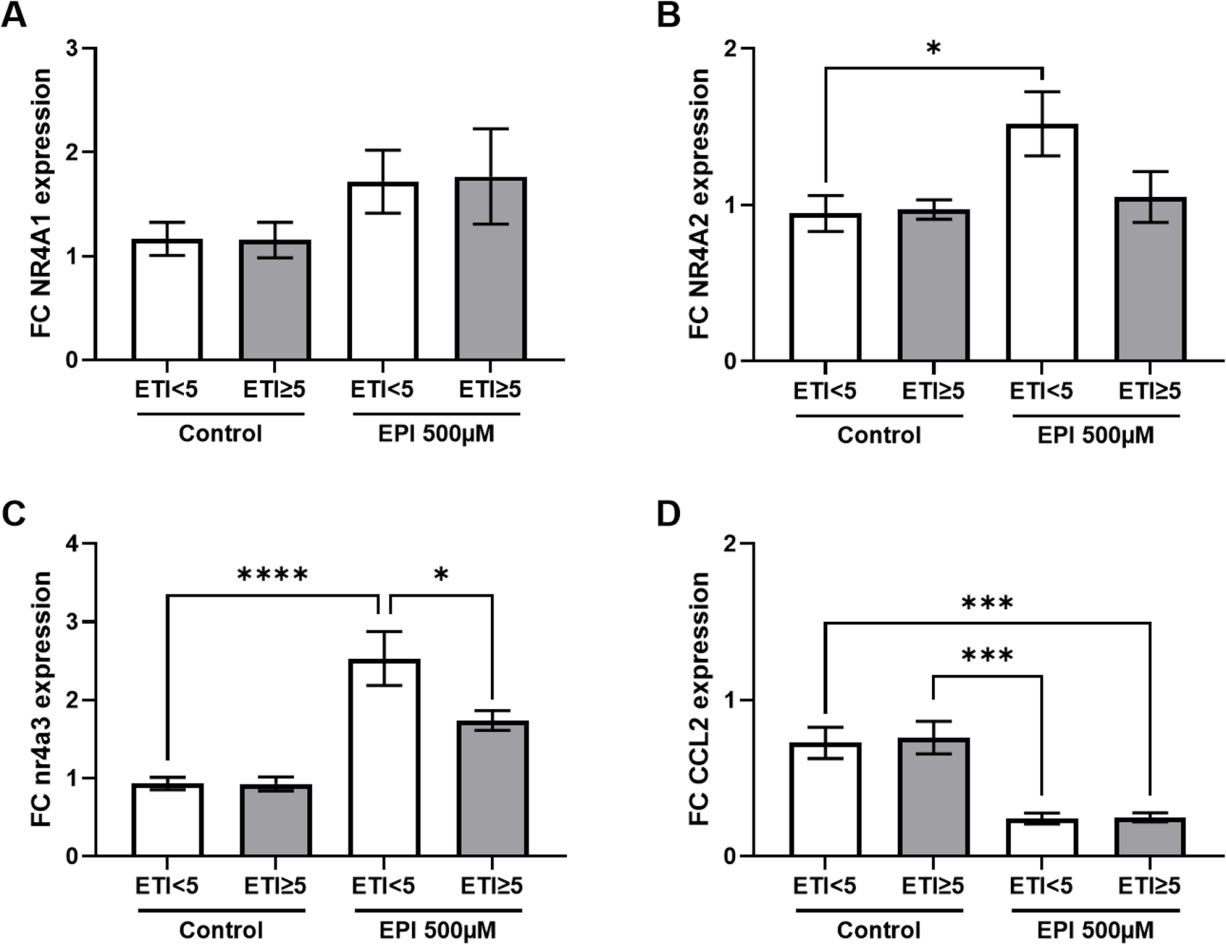
Blunted high-dose epinephrine-induced upregulation of PBMC nuclear receptor expression in ELS patients. Fold-change (FC) nuclear receptor 4 a 1 (NR4A1) (**A**), NR4A2 (**B**), and NR4A3 (**C**) mRNA expression per gapdh between resting phase A and resting phase B in peripheral blood mononuclear cells (PBMCs) after 1 h of stimulation with either control medium (Control) or 500 µM epinephrine (EPI) from patients with coronary heart disease and a sum score < 5 vs. ≥ 5 in the Early Trauma Inventory self-report short-form (ETI). FC cc-chemokine-ligand-2 (CCL2) mRNA/gapdh expression in the same groups as above (**D**). N(A Control ETI < 5) = 14, N(A Control ETI ≥ 5) = 13, N(A EPI ETI < 5) = 16, N(A EPI ETI ≥ 5) = 12, N(B Control ETI < 5) = 15, N(B Control ETI ≥ 5) = 11, N(B EPI ETI < 5) = 17, N(B EPI ETI ≥ 5) = 13, N(C Control ETI < 5) = 14, N(C Control ETI ≥ 5) = 11, N(C EPI ETI < 5) = 15, N(C EPI ETI ≥ 5) = 9, N(D Control ETI < 5) = 14, N(D Control ETI ≥ 5) = 12, N(D EPI ETI < 5) = 12, N(D EPI ETI ≥ 5) = 11, ANOVA, *p < 0.05, ***p < 0.005, ****p < 0.001

We observed no FC expression increase in PBMCs from ETI ≥ 5 patients with respect to NR4A1 (Fig. 5A), NR4A2 (Fig. 5B), or NR4A3 (Fig. 5C) in control medium. However, upon high-dose epinephrine the expression of nuclear receptors NR4A1, -2, and NR4A3 was increased. This was most significant in NR4A2 and NR4A3 in patients with reported low early life stress (ETI < 5) as opposed to patients who reported early life stress (ETI ≥ 5). In the case of NR4A3 EPI-induced upregulated FC of expression was markedly blunted in ELS patients, suggestive of potential NR4A3-driven inhibition of pro-inflammatory gene expression (Fig. 5C). CC-chemokine-ligand 2 (CCL2) expression was markedly blunted upon EPI (Fig. 5D).

## Discussion

We conducted an observational study comparing coronary heart disease patients with and without self-reported significant early life stress (ELS) set apart by a median split of the population’s ETI sum score. Here, we show that IL-6 cytokine release from PBMCs isolated from ELS patients is significantly higher than of control patients even in the absence of psychological comorbidity like PTSD. Furthermore, there seems to be a dose dependent effect of ELS, as a significant increase in IL-6 at resting phase B, as well as fold-change values to recovery for IL-1β across the ETI-SR-SF sum score could be found. Stimulation with a high dose of epinephrine to simulate excessive stress blunted IL-6 release in ELS patients, implicating catecholamine-independent pathways in the latter. Interestingly, epinephrine stimulation triggered a significant upregulation of nuclear receptor expression in PBMCs as opposed to IL-6 downregulation, particularly of NR4A3. This effect was markedly ameliorated in PBMCs from ELS patients potentially hinting at a connection, since NR4A3 has been shown to inhibit nuclear factor kappa B (NF-kB) with suppression of the inflammatory response [19, 31]. This may in part contribute to the increased cardiovascular risk of ELS patients [32].

**Fig. 6:**
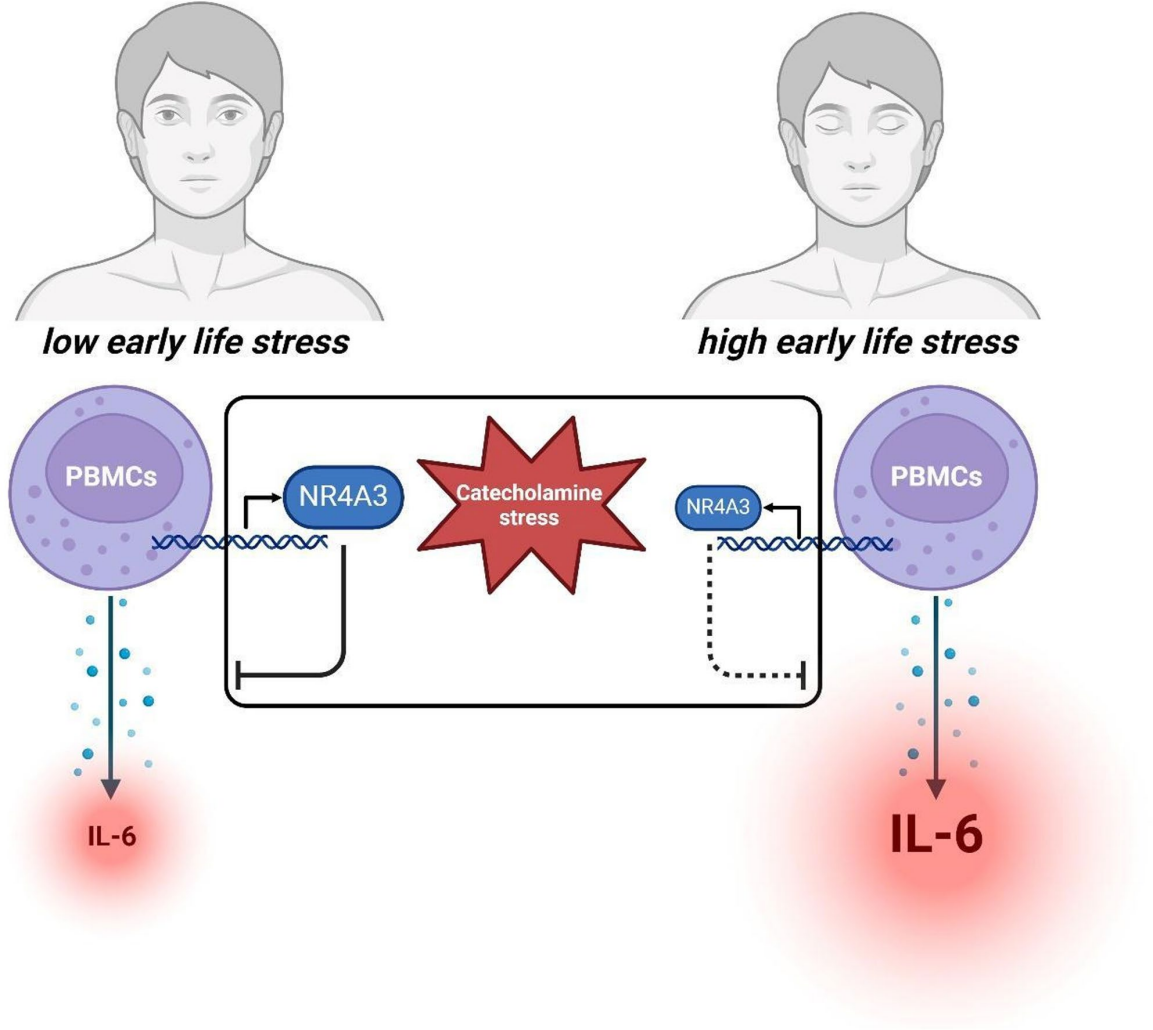
Working model of peripheral blood mononuclear cell-driven IL-6 release in coronary heart disease patients reporting low vs. high early life stress. In our data patients with high self-reported early life stress show significantly higher release of interleukin-6 (IL-6) by peripheral blood mononuclear cells (PBMCs) after a walking challenge ex vivo. Additional stimulation of PBMCs with a high dose of epinephrine to mimic excessive catecholaminergic stress showed a trend of reduction of PBMC IL-6 release, while indicating a significant upregulation of nuclear receptor expression in patients reporting low early life stress, particularly of the nuclear receptor 4A3 (NR4A3). This effect was less pronounced in patients suffering from high early life stress, suggesting a potential anti-inflammatory contribution of nuclear receptor expression in PBMCs

In summary, our findings point to a potential involvement of PBMC-dependent signaling in CHD among patients with ELS and indicate that this may be related to the increased cytokine release observed in PBMCs from CHD patients with significant ELS compared to those without (Fig. 6).

Regarding baseline patient characteristics in our small cohort, we did not observe significant differences, including inflammatory markers like CRP or procalcitonin. Still, CRP levels in the ETI > 5 group were approximately twofold higher than in controls, albeit not yet clinically relevant and potentially indicative of elevated chronic inflammation. Of note, we observed a significantly higher resting heart rate in ELS patients, which is in line with findings from other studies regarding adverse childhood experiences [33] and was shown to be associated with an increased risk of death from cardiovascular and non-cardiovascular diseases [34]. Also, pulmonary hypertension was significantly more prevalent in ELS group potentially influencing severity of CHD. In saliva we measured levels of oxytocin, alpha amylase (sAA), and cortisol after the first resting phase. As outlined above, cortisol and oxytocin are surrogate parameters and modifiers of the HPA axis. Particularly sAA is considered a non-invasive marker of sympathetic activity that is elevated by psychosocial stress [35]. In summary, we did not observe any significant differences regarding salivary oxytocin, cortisol or sAA. This might be explicable by our small sample size or the timing of saliva collection [36]. Nevertheless, we observed a trend of reduced sAA and cortisol in ELS patients. In line with these findings, lower levels or blunted diurnal profiles of sAA were found in family caregivers for patients with brain cancer and dementia, subjects exposed to chronic stress [36–38], as well as in subjects exposed to early life adversity [12, 39]. Our finding of a trend towards reduced baseline salivary cortisol in ELS patients is also in line with the literature showing a negative correlation between trauma and baseline cortisol [39]. In this regard, hypocortisolism in PTSD patients has been suggested to be causative of elevated low grade inflammation [40]. And dysregulation of HPA activity has been shown to be associated with cardiovascular diseases, including hypertension and CHD [41].

In PBMCs from patients with CHD and ELS our data shows a significant increase in IL-1β expression and a significantly release of IL-6 over 24 h compared to the control group. For IL-1β this effects persists after multivariate hierarchical regression analysis controlling for sex, hyperlipidemia, diabetes, obesity, and NT-proBNP. The same could not be shown for IL-6 which we assume might be due to overfitting given our small sample size. These findings are in line with Gola et al. showing that spontaneous production of IL-1β and IL-6 by isolated PBMCs of patients with PTSD were higher than those of the control group [14]. Interestingly they found no difference in plasma serum levels of forementioned cytokines, analogous to our data where there was no difference in serum proinflammatory markers (leucocytes, CRP, procalcitonin, Ferritin) as well. Conversely, after social stress (Trier Social Stress Test) Carpenter et al. observed elevated serum IL-6 in an ELS group compared to control [42]. This discrepancy may be explicable by the lack of repetitive measurements or acute psychosocial stress in our study. When looking at the general population with participants followed up from birth to age 32, Danese et al. observed an association of ELS with inflammation and cardiovascular risk as defined by the American Heart Association (high-sensitive CRP > 3 mg/L). They attributed more than 10% of the inflammation to ELS alone [43]. In our study epinephrine stimulation ex vivo showed a trend to suppress IL-6 expression and release from PBMCs. In CHD following early childhood stress, this seems to be associated with inflammation that might be independent of acute catecholaminergic stimulation. Our finding of elevated nuclear receptor expression, blunted in PBMCs from ELS patients may suggest discrepant PBMC signaling during catecholaminergic stimulation in ELS. In this regard, NR4A3 has been shown to inhibit inflammation by NF-kB suppression [44, 45].

There are several limitations to our study. First, our small sample size as well as the cross-sectional design with retrospective investigation of childhood adversities may lead to bias and prevents causal inference. Hence, our conclusions need to be interpreted carefully, generalizability is limited, and further investigation in larger trials is needed. Furthermore, dichotomization of the ETI total score reduces statistical power and obscures variability. Our decision to dichotomize was driven by both conceptual and pragmatic considerations. Specifically, the primary aim of the study was to contrast individuals with clinically meaningful exposure to early trauma versus those without such exposure, consistent with prior literature that conceptualizes early trauma as a categorical risk factor rather than a purely dimensional construct. Moreover, given the relatively small sample size, modeling ETI as a continuous predictor while simultaneously adjusting for multiple covariates risked overfitting und unstable parameter estimates. Importantly, the ETI cut-off used in the present study was based on established conventions in prior work, enhancing interpretability and comparability with existing findings. Our salivary samples lack sequential measurements to create individual diurnal profiles of sAA, cortisol and oxytocin which substantially limits interpretation. Primarily, in our study this has to do with difficulties in collecting saliva from elderly patients and structural constraints working with study participants in a medical setting on a psycho-cardiology ward. The latter also carries the possibility of confounding by overall stress due to hospital environment. Furthermore, we found no differences in our psychometric diagnostics regarding psychological stress (PHQ) in between ELS and no-ELS group which might explain null findings in saliva samples. A non-significant trend towards higher stress and anxiety scores could be observed in ELS group as possible confounder despite a priori exclusion of patients diagnosed with psychiatric disorders. Finally, 5 out 15 patients in ELS group were on L-thyroxine compared to none in the control group which carries a risk for bias by immunomodulating effects. Additionally, as the ETI-SR-SF relies on retrospective self-report, the assessment may be subject to recall bias and current mood-dependent reporting. However, retrospective measures of childhood adversity have shown acceptable reliability in adult populations [22, 24, 25].

Taken together, our data indicate that even in the absence of diagnosed PTSD, PBMCs of CHD patients with self-reported ELS are prone towards elevated cytokine expression and release. Also, our data indicate HPA axis hypoactivity upon ELS, potentially causing a pro-inflammatory priming due to lack of corticoid baseline suppression. One possible pathway through which this might be mediated is NF-κB: Early life stress has been shown to cause epigenetic upregulation of FKBP5 inducing NF-κB–driven peripheral inflammation (IL-1 and IL-6 among others) and thereby heightening cardiovascular risk [46] by promoting atherosclerosis [47]. This heightened NF-κB driven peripheral inflammation is what we might have seen measuring IL-1β expression and IL-6 release in PBMCs and is also in line with findings from Carpenter et al., who found a positive association between plasma IL-6 response to a psychosocial stressor and self-rating on the childhood trauma questionnaire [42]. In line with the clinical and stratified medical perspective [48] our findings may enrich the debate on whether childhood adversities could serve as environmental marker for distinguishing significant clinical subgroups within CHD patients, as shown elsewhere [11, 49–51].

## Conclusions

Our data and the studies discussed support the impact of early childhood adversity on systemic PBMC-driven inflammation later in life. The clinical implications for our study lie in a need for psychosocial screening in cardiac patients, which should include early life adversity. Also, a broad spectrum of therapeutic approaches including mindfulness based interventions [52] as well as anti-inflammatory treatments [53–56] and laboratory diagnostic approaches [57] are under investigation in CHD patients. Finally, low grade inflammation in CHD ELS patients, appears to be at least in part driven by PBMCs. Further studies are necessary to dissect the role of PBMCs in patients with early childhood adversity.

## Data Availability

Data will be made available upon reasonable request.

## Abbreviations

CHD: Coronary heart disease
ELS: Early life stress
ETI-SR-SF: Early trauma inventory self report short form
HPA: Hypothalamic-pituitary-adrenal
IL-1β: Interleukin-1β
IL-6: Interleukin 6
NR4A3: Nuclear receptor 4A3
PHQ-D: Patient health questionnaire
PBMCs: Peripheral blood mononuclear cells
PTSD: Posttraumatic stress disorder
SF-36: Short form-36 health survey
SNS: Sympathetic nervous system
